# Hypogonadotropic hypogonadism due to compound heterozygous mutations TACR3 in siblings

**DOI:** 10.1002/ccr3.3370

**Published:** 2020-09-22

**Authors:** Rita Valsassina, Filipa Briosa, Joana Soares, Marta Amorim, Catarina Limbert

**Affiliations:** ^1^ Department of Pediatrics Hospital Beatriz Ângelo Loures Portugal; ^2^ Department of Pediatrics Hospital de Santo André Leiria Portugal; ^3^ Department of Genetics Hospital Dona Estefânia Lisboa Portugal; ^4^ Department of Pediatric Endocrinology Hospital Dona Estefânia Lisboa Portugal

**Keywords:** endocrinology and metabolic disorders, genetics, pediatrics and adolescent medicine

## Abstract

The authors present a new association of two heterozygous TACR3 mutations (p.Arg230His and p.Trp275*) responsible for a clinical trait of normosmic congenital hypogonadotropic hypogonadism in a family.

## Graphical abstract

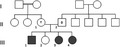

## 1 INTRODUCTION

Congenital hypogonadotropic hypogonadism (HH) has an association to hereditary causes in 30%‐50% of affected subjects. TACR3 is one of the genes with a higher number of known mutations. The authors present a new association of two heterozygous TACR3 mutations, found in three siblings diagnosed with normosmic HH.

Puberty is a specialized phase in human development characterized by a gradual increase in the hypothalamo‐pituitary‐gonadal axis’ activity during the second decade of life and which lasts from two to five years.^1^ The hallmark of puberty is the beginning of a pulsatile secretion of gonadotropin‐releasing hormone (GnRH) by hypothalamus, which triggers the release of the gonadotropin luteinizing hormone (LH) and follicle‐stimulating hormone (FSH) from the pituitary gland.^2^ These hormones act in gonadal tissue to drive both sex hormone secretion and gametogenesis, therefore inducing maturation of secondary sexual features and reproductive system.^1^, ^2^

In hypogonadotropic hypogonadism (HH), there is a failure of puberty onset due to insufficient gonadotropin release for unknown reasons. This results in the incapacity to develop secondary sexual characteristics and a mature reproductive system at 13 years of age in girls and 14 years of age in boys.^1^, ^3^ Traditionally, HH is divided into two categories: Kallmann syndrome (KS) and normosmic HH (nHH), based on the absence or presence of normal sense of smell, respectively.^1^, ^3^, ^4^, ^5^

Hypogonadotropic hypogonadism can be inherited or acquired. Currently, known genetic defects account for about 30%‐50% of all HH cases with around 50 mutated genes already associated.^1^, ^3^, ^5^ Loss‐of‐function TACR3 mutations have been described as causative of congenital HH, pointing out the key role of this gene in the regulation of the human gonodotrope axis.^6^

## 2 CASE REPORT

**Figure 1:**
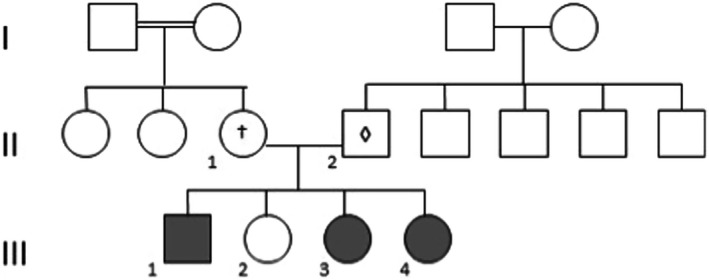
Family Pedigree. Solid symbols indicate affected subjects (III‐1, III‐3, III‐4), and open symbols indicate subjects with no mutations. The symbols ⋄ and † indicate unaffected heterozygotes. ‐ ⋄ ‐ refers to the mutation c.824G>A (p.Trp275*) inherited from the subject II‐2 and † to the mutation c.689G > A (p.Arg230His) from subject II‐1. Circles represent female family members and squares male family members

In a family originated from Lisbon (Portugal), three of four children of nonconsanguineous parents were found to have nHH (Figure 1).

The index case was a young man (Subject III‐1) who was referred at 16 years of age because pubertal development had not occurred. He had typical signs of complete hypogonadism, with small intrascrotal testes (2‐3 ml), spar pubic hair (P1‐2), and a small phallus. His height was 161.5 cm (*Z*‐score −1.57), his weight 61.9 kg (*z*‐score 0.08), and his bone age 12.5 years. His body proportions were normal. He had a normal sense of smell, and no renal or craniofacial abnormalities.

His affected sisters (Subjects III‐3 and III‐4) were evaluated at 14 and 13 years old, respectively, and also presented complete hypogonadism and a normal sense of smell on olfactometry. They presented with no breast development, no pubic hair, and no menarche. Subjects III‐3 and III‐4 heights were 153 cm (*z*‐score −1.19) and 144.2 cm (*z*‐score −2.04), respectively, their weights were 51.3 kg (*z*‐score 0.53) and 43.1 kg (*z*‐score 0.15), and their bone ages 12.5 and 10 years. Their developmental background was normal. There was no history for eating disorder, chronic illness, or head trauma.

The unaffected sister (Subject III‐2) had normal pubertal development with spontaneous regular menses at 13 years old.

The onset of their mother's (Subject II‐1) menarche was at 15 years old. She reported having had spontaneous regular menses, unassisted conception, and normal pregnancies. Their father (Subject II‐2) stated that he had a normal pubertal development at 13 years old.

All the unaffected family members presented a normal sense of smell.

Hormone assays revealed very low plasma testosterone levels in the affected brother and an undetectable plasma estradiol level in the affected sisters (Table 1). Very low plasma gonadotropin levels were detected in all the subjects.

**Table 1:** Data for affected family members

Subject	III‐1	III‐3	III‐4
Age (y)	16	14	13
Sense of smell	Normal	Normal	Normal
Breasts (Tanner stage)	I	I	I
Pubic hair (Tanner stage)	I‐II	I	II
External genitalia (Tanner stage)	I	I	I
Penis length (cm)	4	‐	‐
Testicular volume (mL)	2‐3	‐	‐
LH (mIU/mL)	0.2	<0.1	<0.09
FSH (mIU/mL)	0.6	0.7	0.29
Estradiol (pg/mL)	ND	<10	<10
Testosterone (ng/mL)	0.14	0.15	ND
Prolactin (ng/mL)	3.42	4.3	ND
IGF‐1 (ng/mL)	272	144	144
Bone age (y)	12.6	12.5	10
MRI head	Normal	Normal	ND
LH pulsatility	Apulsatile	ND	ND

Note

Normal ranges for the hormones listed are as follows: for men: FSH, 0.95‐11.95 mIU/mL; LH, 0.57‐12.09 mIU/mL; testosterone, 3‐12 ng/mL; prolactin, 4.2‐23.04 ng/mL; and IGF‐1, 163‐584 ng/mL for Tanner stage I; for women (in early follicular phase): FSH, 3.03‐8.08 mIU/mL; LH, 1.8‐11.78 mIU/mL; estradiol, 21‐251 pg/mL; prolactin, 11‐23 ng/mL; and IGF‐1, 71‐394 ng/mL for Tanner stage I. ND denotes not done.

All of them had normal function of the anterior pituitary, thyroid, and adrenal glands, along with normal levels of ferritin and serum insulin‐like growth factor I. Subjects III‐1 and III‐3 presented normal findings on MRI of the pituitary and olfactory bulbs, and subject III‐4 is still awaiting for the completion of her study. The affected male presented an apulsatile result in endogenous basal LH pulsatility study.

Pelvic sonography revealed an absence of uterus and the presence of two small ovaries (right, 13 mm; left 22 mm), with no visible follicles, in subject III‐3. In subject III‐4, it showed a small uterus (22 mm) and two small ovaries (right, 19 mm; left 13.7 mm), with no visible follicles.

Karyotypes were normal in index case subject III‐1 (46,XY) and subjects III‐3 and III‐4 (46,XX).

A diagnosis of nHH was established based on the absence of spontaneous puberty and hypogonadal sex steroid levels (testosterone ≤ 100 ng/dL; oestradiol ≤ 20 pg/mL) at a normal pubertal age in all three subjects in the setting of inappropriately low gonadotropin levels without associated anosmia and no evidence of a mass lesion on the imaging of hypothalamic pituitary region. Normal basal levels of thyroid‐stimulating hormone, prolactin, and adrenal hormones supported the diagnosis.

Genetic study in the index case revealed two heterozygous mutations on TACR3 gene: c.824G>A (p.Trp275*), previously described as pathogenic, and c. 689G>A (p. Arg230His), with conflicting evidence about its pathogenicity. The TACR3 gene is responsible for autosomal recessive hypogonadotropic hypogonadism type 11, with or without anosmia. The studies to the parents confirmed the compound heterozygosity, and the studies performed to the siblings confirmed segregation of the genotype with the phenotype: Case III‐3 and III‐4 shared the two TACR variants with index case. These results confirmed the pathogenicity of the second variant (p. Arg230His) and the clinical diagnosis.

The index case received testosterone enanthate treatment at 16.5 years for 3 months, with adequate virilization response. Three months later, the testis volume was 3‐4 mL, so gonadarche was induced with human chorionic gonadotrophin starting from a dose of 1500 UI twice a week for 6 months. During puberty induction, testis volume increased to 6 mL and a progressive rise of testosterone and inhibin B was observed. During the next four years, he progressively gained testicular volume (testes 12‐15 mL) and penis length (9 cm stretched length) in a spontaneous manner. At 21 years old, he presented a Tanner stage of P5G4A4.

Subject III‐3 started continuous transdermal low‐dose conjugated estrogen with a confirmed onset of breast growth. After 8 months of treatment, control pelvic sonography revealed an uterus (65 × 15 mm, 7 × 15 mm) with centered endometrium and the presence of two ovaries (right 24.3 × 15 mm; left 15 × 16.5 mm) with small follicles.

Subject III‐4 will start continuous transdermal low‐dose conjugated estrogen.

All subjects are on regular observation and follow‐up at a pediatric endocrinology outpatient clinic.

## 3 DISCUSSION

Congenital HH is a very rare genetic disorder characterized by complete or partial lack of pubertal development therefore leading to infertility associated to complete or partial absence of GnRH‐mediated release of LH and FSH in the setting of otherwise normal anterior pituitary anatomy and function.^1^, ^3^, ^4^, ^5^

According to recent knowledge, only one‐third of the HH cases have an inheritable basis. However, the autosomal recessive mode of inheritance accounts just for 30% of these familial cases.^4^ Genetic defects of GNRHR, GNRH1, KISS1R, and TAC3/TACR3 are implicated in HH. Mutations in TAC3/TACR3, as seen in this family, have been reported in 5% cases of HH inherited by the autosomal recessive mode.^1^, ^3^, ^4^, ^5^, ^6^

TAC3 encodes Neurokinin B (also known as tachykinin neurokinin‐3), a neurotransmitter, which is primarily expressed by hypothalamic neurons. This neuropeptide is the ligand for the neurokinin‐3 receptor, which is encoded by TACR3 gene.^7^

The mutations of both genes are associated with a Neurokinin B malfunction. Precise mechanisms by which these mutations cause gonadotropin deficiency and HH are not yet clear; however, these mutations have a critical role in sexual maturation.^6^, ^8^

The mutation 824G>A (p.Trp275*) in TACR3 is one of the mutations described in this family and produces a stop codon in the 5th transmembrane segment (p.Trp275Stop) of NK3R, resulting in an absent or disrupted protein product with a pathological effect.^6^, ^9^ The second identified mutation in this family (c.689G>A (p.Arg230His)) involves the alteration of a nucleotide that is conserved through species (Arg230) producing a variant currently classified of uncertain significance due to lack of clinical information and functional studies.^10^

The genetic study of this family favors the pathogenicity of the second variant (p. Arg230His), since all three individuals with the compound heterozigosity present with the clinical diagnosis and all three familial members that are heterozygous for a single mutation (subjects II‐1, II‐2 and III‐2) have normal pubertal development, regardless of gender (complete genotype‐phenotype correlation).

The treatment of nHH is primarily focused on hormone replacement with sex steroids (testosterone, estrogen, and progesterone) in order to promote normal sex hormones’ levels, the development of secondary sex characteristics, and a eugonadal state. GnRH or gonadotropin therapies are the best options for the infertility treatment.^11^, ^12^, ^13^

After discontinuation of sex steroid therapy, patients with TAC3/TACR3 mutations sometimes show partial sexual maturation and often experience partial or complete recovery of their reproductive axis with spontaneous fertility achieved in some cases.^1^, ^2^, ^5^, ^7^, ^14^, ^15^, ^16^ At 21 years of age, the affected male reached Tanner stage IV‐V after 9 months of puberty induction with testosterone and human chorionic gonadotrophin, whereas his 17‐year‐old sister is still on transdermal steroid replacement with Tanner stage III and no menarche. A longer follow‐up of this family is required to understand whether sexual maturation and fertility will be achieved in all three siblings.

This familial case of congenital HH seems to be a unique disease model in which a new described association of two heterozygous TACR3 mutations (p.Arg230His and p.Trp275*) appears to be responsible for a clinical trait.

Further genetic testing considering these compound heterozygous mutations may therefore play a key role in the diagnosis and prognosis of nHH, opening barriers to novel disease classification and unraveling new paths for gene therapy in the future.

## CONFLICT OF INTEREST

None declared.

## AUTHOR CONTRIBUTIONS

RV, FB, and JS: wrote and designed the primary manuscript. MA and CL: responsible for the critical revision concerning Genetics and Pediatric Endocrinology, respectively, and for the approval of the final version.

## ETHICAL APPROVAL

All the subjects involved in this clinical case provided consent for the publication of this case report.
